# Folate contents in insects as promising food components quantified by stable isotope dilution

**DOI:** 10.3389/fnut.2022.970255

**Published:** 2022-09-08

**Authors:** Nadine Weber, Lenka Kouřimská, Martin Kulma, Dora Petříčková, Franziska Seufert, Michael Rychlik

**Affiliations:** ^1^Chair of Analytical Food Chemistry, Research Department Life Science Engineering, TUM School of Life Sciences, Technical University of Munich, Freising, Germany; ^2^Department of Microbiology, Nutrition and Dietetics, Czech University of Life Sciences Prague, Praha-Suchdol, Czechia; ^3^Department of Zoology and Fisheries, Czech University of Life Sciences Prague, Praha-Suchdol, Czechia; ^4^Centre for Nutrition and Food Sciences, Queensland Alliance for Agriculture and Food Innovation, The University of Queensland, St Lucia, QLD, Australia

**Keywords:** vitamin B, edible insects, LC-MS/MS, novel food, insect feed

## Abstract

Concerning the increasing population, edible insects are of growing interest due to several advantages such as sustainable production and as a source of high-quality nutrients. One of the less studied nutrients are folates, in the context of insects is folates, which play an important role in human metabolism. In the article, we describe how six different insect species are reared and extracted for five common folate vitamers by high-performance liquid chromatography interfaced with tandem mass spectrometry (LC-MS/MS). For this purpose, house crickets (*Acheta domesticus*—adults), Jamaican field crickets (*Gryllus assimilis*—adults), discoid cockroaches (*Blaberus discoidalis*—nymphs), migratory locusts (*Locusta migratoria*—adults), mealworms (*Tenebrio molitor*), and lesser mealworms (*Alphitobius diaperinus*) were investigated. The total folate content differs between 59.1 ± 6.50 and 143 ± 11.1 μg/100 g. Also, the feed, which was adapted to the needs of the insects and differed for some species, was extracted for their total folate content. The four different feed compositions (rapeseed, chicken feed, bramble leaves, and a mix of chicken feed, wheat bran, and carrot/apples) showed a folate content of about 100 μg/100 g, except for hay, where the content was 300 μg/100 g. In general, the insect folate content is comparable to other food and seems to be a promising source of folates. However, the amount of consumption needed to meet the requirement must also be considered. Moreover, the effect of different influencing factors is not yet entirely clear and needs further investigation.

## Introduction

In the first 2 decades of the twenty first century, the increasing world population, global climate change, and unsustainability of agriculture sector production were the main drivers leading to the intensification of research on alternative nutrient sources (1). Moreover, the recent outbreak of the COVID-19 pandemic in 2020 reminded the risks connected with zoonotic viral epidemics, which can also be associated with livestock farming (2), and thus highlighted the urgent need for the development of a strategy on how to obtain novel protein sources. Moreover, “sourcing and developing new protein alternatives” is one of the goals of FOOD 2030 EU policy (3). Based on recent knowledge, insects appear to belong to the most promising food and feed protein alternatives. Compared to conventional sources, the main benefits of edible insects are lower greenhouse gas emissions, lower requirements of space and water, and higher efficiency of feed conversion (4). Also, they might be cultivated on food industry by-products or biowaste (5–7). Therefore, the artificially reared insects seem to have the potential to become both environmental-friendly and feasible components of foods and feeds.

With regard to current legislation on insects as food, Regulation (EU) 2015/2283 on novel foods stated that the categories of novel foods should cover whole insects and their parts. This regulation has been applicable since 1 January 2018. According to this regulation, each insect species must go through an approval process before it can be included in the list of novel foods by the commission and can be placed on the market (8).

On 24 November 2020, the European Food Safety Authority (EFSA) issued its first opinion on dried larvae of *Tenebrio molitor* (9). In its opinion, the EFSA panel noted that the consumption of dried *T. molitor* larvae is not nutritionally disadvantageous and that based on the toxicological studies submitted, there is no concern for the safety of this food. Other opinions issued by the EFSA on 25 May and 7 July 2021 were on frozen and dried formulations of *Locusta migratoria* (10), *Acheta domesticus* (11), and *T. molitor* larvae (12). Considering toxicity studies from the literature and the history of use, the EFSA panel had no concerns identified, apart from allergies to crustaceans and dust mites such as *A. domesticus*, *L. migratoria*, and *T. molitor* larvae. For *A. domesticus* and *L. migratoria*, additionally, an allergy to mollusks can occur. In the near future, the EFSA is expected to publish an opinion on other insect species.

From the nutritional point of view, the chemical composition of insects is known to be influenced by many factors, including species, developmental stage, rearing technology, and diet composition (13–15). Generally, insects are rich in proteins and lipids, whose quality is comparable to traditional protein sources (16–18). While macronutrients of the majority of edible species have been recently analyzed by multiple authors, the reports on minor nutrients such as some minerals and vitamins are relatively scarce. Although clear conclusions on this topic could not be drawn especially due to limited research data using different approaches of sample preparations and analytical methods, the results of some authors indicate that insects appeared to be a very good source of vitamins (13, 19, 20). Regarding water-soluble vitamins, insects appeared to be high in B vitamins, with exception of B1 (14, 21).

Among water-soluble vitamins, folates play a particular role in human metabolism. They are essential as coenzymes for one carbon transfers and synthesis of nucleotides and amino acids (22, 23). As folates cannot be synthesized by the human body, they must be supplied with food or supplements. In general, for many countries, a suboptimal supply of folates is assumed, and in particular, for those, fortification with folic acid is not mandatory (24). The sufficient intake of folates is particularly relevant for young women of childbearing age since a deficiency is associated with an increased risk for neural tube defects in newborns (25). A suboptimal intake of folate may also cause chronic diseases such as Alzheimer’s, autism, and cardiovascular disease (26–29).

Folates being such a critical group of vitamins, there has been recent research to elucidate promising folate sources in foods or food components. In this regard, for example, some berries and exotic fruits, yeast and yeast extracts, and algae have been found to contain significant folate contents to cover at least partly the recommended daily intake.

The investigated vitamers were folic acid (PteGlu), tetrahydrofolate (H_4_folate), 5-methyltetrahydrofolate (5-CH_3_-H_4_folate), 5-formyltetrahydrofolate (5-CHO-H_4_folate), and 10-formylfolic acid (10-CHO-PteGlu). One of the most important vitamer is certainly 5-CH_3_-H_4_folate. It is the active form in the body and can serve as a marker for the folate status in blood plasma. Also, in fruits and vegetables, 5-CH_3_-H_4_folate and 5-CHO-H_4_folate are detected as main vitamers. So far, there is extensive knowledge about the metabolization of PteGlu, H_4_folate, 5-CH_3_-H_4_folate, and 5-CHO-H_4_folate, but the role of 10-CHO-PteGlu in metabolism has not yet been finally clarified (30, 31).

For food folate analysis, liquid chromatography–mass spectrometry (LC-MS/MS) coupled with stable isotope dilution assay (SIDA) is increasingly becoming the method of choice. The use of a stable isotopically labeled analog as the internal standard compensates for analyte degradation, analyte conversion, or losses during extraction as well as matrix effects and ion suppression during LC-MS/MS measurements (32). As compared with traditionally applied microbiological assay (33), SIDA additionally holds the advantage of specifically quantifying single folate vitamers, which allows for predictions about folate stability and bioavailability (34). In this study, a broad range of insects were analyzed regarding their total folate content and vitamer profiles, along with their feed, to allow for drawing respective correlations. All samples were analyzed by SIDA and LC-MS/MS according to the method mentioned in a previous work (35).

To the best of our knowledge, there are very limited data about the folate content in insect species that are commonly used as feed and increasingly as a food constituent. Therefore, the aims of our study were (1) to analyze the insects for their folate content, (2) to investigate the possible parameters influencing the folate content, and, finally, (3) to assess the insects as a promising source for folates in the human diet.

## Materials and methods

### Cultivation of insects

**FIGURE 1:**
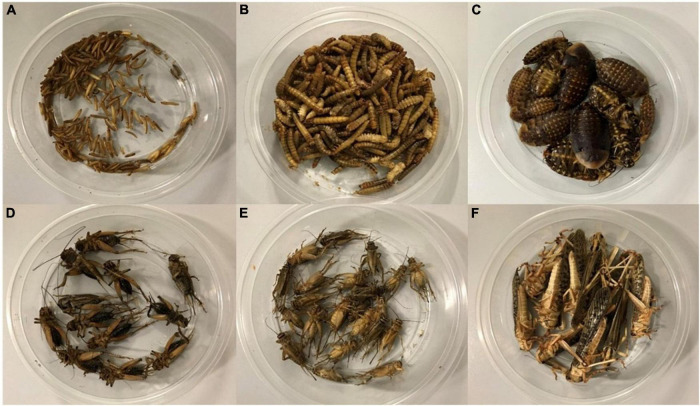
Analyzed insect species *A. diaperinus* **(A)**, *T. molitor* **(B)**, *B. discoidalis* **(C)**, *A. domesticus* **(D)**, *G. assimilis* **(E)**, and *L. migratoria* **(F)**.

The insects for the analysis (Figure 1) were obtained from the experimental colonies reared in the insectarium at the Faculty of Agrobiology, Food, and Natural Resources, Czech University of Life Sciences Prague, at 27 ± 1°C and 40-50% relative humidity, and 12:12 photoperiod using a rack system. A 40-W bulb, in the same regime as the artificial lightning (12:12), was used as an additional heat source for *L. migratoria*. The insects were fed *ad libitum* on chicken feed (CH) (wheat 78.0%, soybean meal 17.6%, rapeseed oil 1.8%, limestone 1.0%, monocalcium phosphate 0.7%, vitamin premix 0.5%, sodium carbonate 0.4%, salt 0.1%), which is commonly used as a standard or a control feed for insects [e.g., (13)]. One sample of *A. domesticus* was provided by modified chicken feed containing rapeseed meal (RP) as the main protein component (rapeseed meal 70.0%, wheat 21.5%, rapeseed oil 5.3% limestone 1.0%, monocalcium phosphate 0.7%, vitamin premix 0.5%, sodium carbonate 0.4%, salt 0.1%). In the case of *A. diaperinus* and *T. molitor*, the colonies were kept on a substrate formed by wheat bran (WB) and CH in a 4:1 ratio. Finally, CH in the colony of *L. migratoria* was daily supplemented by fresh bramble leaves (BL) and hay. Regarding water sources, water gel (Oslavan, Náměšt’ nad Oslavou, Czech Republic) was provided to the insects, with exception of both mealworms. For them, slices of apples and carrots were used for this purpose. Prior to analysis, the insects were segregated from all feed sources, fasted for 24 h, harvested (harvest instar: *A. diaperinus* and *T. molitor*: larvae—harvested when the first pupae occurred; *B. discoidalis*—pre-subadults and subadults; *A. domestica*, *G. assimilis*, and *L. migratoria*—adults), freeze-killed, and frozen-stored at –80°C. All samples were transported frozen and stored at –20°C till they were processed. Only the samples of *A. domesticus* (2) were lyophilized [CoolSafe 110-4 (SCANVAC, Denmark, and rotary vane pump FB65460 (ILMAC, Germany)] and homogenized by using a laboratory mill (Grindomix 200, Haan, Germany) before their transportation.

### Chemicals and solutions

#### Chemicals

Acetonitrile, methanol, and water were purchased by Honeywell (LC-MS grade) (Seelze, Germany) and VWR (HPLC grade) (Ismaning, Germany); ascorbic acid, formic acid (≥99%, LC-MS grade), acetic acid (99%, LC-MS grade), sodium hydroxide, and sodium chloride from VWR (Ismaning, Germany); 2-(N-morpholino)-ethanesulfonic acid (MES), dithiothreitol (DTT), and active charcoal were from Sigma-Aldrich (Steinheim, Germany); potassium dihydrogen phosphate and sodium acetate trihydrate from Merck (Darmstadt, Germany); and disodium hydrogen phosphate (anhydrous) from Alfa Aesar (Karlsruhe, Germany).

Rat serum was purchased from Biozol (Eching, Germany), and chicken pancreas was from Difco (Sparks, MD, United States). For solid-phase extraction, Strata^®^ strong anion exchange (SAX) cartridges from Phenomenex (Aschaffenburg, Germany) were used.

The unlabeled (PteGlu, H_4_folate, 5-CH_3_-H_4_folate, 5CHO-H_4_folate, 10-CHO-PteGlu) reference compounds were purchased from Schircks Laboratories (Jona, Switzerland), whereas the labeled (^13^C-PteGlu, ^13^C-H_4_folate, -5-CH_3_-H_4_folate, ^13^C-5CHO-H_4_folate, and ^13^C-10-CHO-PteGlu) reference compounds were from Merck & Cie (Schaffhausen, Switzerland).

#### Solutions

The solutions were prepared as described in a previous work with slight modifications (35).

For the extraction solution, MES (200 mmol/L) and ascorbic acid (114 mmol/L) mixed with DTT (6.5 mmol/L) were adjusted to pH 5 with 5M NaOH. For dissolving the unlabeled compounds, a phosphate buffer (100 mmol/L) was used, composed of a disodium hydrogen phosphate (100 mmol/L) aqueous solution adjusted to pH 7 with a potassium dihydrogen phosphate (100 mmol/L) aqueous solution.

The equilibration buffer for SPE clean-up was prepared by mixing the phosphate buffer (100 mmol/L, pH 7) with distilled water in a 1:10 ratio and adding DTT (1.3 mmol/L).

The elution buffer for SPE clean-up was an aqueous solution containing sodium chloride (856 mmol/L), sodium acetate trihydrate (100 mmol/L), DTT (6.5 mmol/L), and ascorbic acid (56.8 mmol/L).

The enzyme solutions for deconjugation were treated with activated charcoal to lower the intrinsic folate of the enzymes. Therefore, rat serum was set with activated charcoal (25 g/L) for 30 min (RT) before the charcoal was removed completely by membrane filtering (PVDF 0.22 μm, Macherey Nagel, Düren, Germany) the solution. The chicken pancreas solution was prepared by mixing ascorbic acid (10 g/L), phosphate buffer (100 mmol/L, pH 7), and lyophilized chicken pancreas (1 g/L) and adjusted to pH 7 by 5M NaOH. Then, activated charcoal (2 g/L) was added and stirred for 30 min (RT) before the charcoal was completely removed by using a membrane filter (PVDF 0.22 μm, Macherey Nagel, Düren, Germany).

To determine the current concentration of the unlabeled compounds by high performance liquid chromatography with diode-array detection (HPLC-DAD), the stock solution was prepared freshly before every sample extraction. For that, 10 mg of PteGlu was solved in 10 mL phosphate buffer and made up to 100 mL with extraction buffer. Also, 2 mg of H_4_folate, 5-CH_3_-H_4_folate, 5-CHO-H_4_folate, and 10-CHO-PteGlu were weighed separately, dissolved in 3 mL phosphate buffer, and made up to 10 mL with extraction buffer. For the measurement on the HPLC, the four solutions containing 200 μL extraction buffer; 400 μL PteGlu, which was used as an internal standard; and 400 μL of H_4_folate, 5-CH_3_-H_4_folate, 5-CHO-H_4_folate or 10-CHO-PteGlu were prepared. To calculate the accurate concentration of the labeled standards, the stock solutions of the unlabeled compounds were diluted at 1:10 for PteGlu and 1:20 for the others and mixed in a respective amount with the ^13^C-labeled compounds for LC-MS/MS response.

### Sample preparation

The samples were homogenized in a grinder (Rommelsbacher, Germany) directly before extraction. The procedure was performed as described in a previous publication with slight modifications (35). All samples were extracted under subdued light and were analyzed in at least duplicates. Therefore, 150 mg of the sample was weighed and was set with a 10-mL extraction buffer with MES. After a 15-min equilibration, a respective amount of ^13^C-labeled standards (^13^C-PteGlu, ^13^C-H_4_folate, ^13^C-5-CH_3_-H_4_folate, ^13^C-5CHO-H_4_folate, and ^13^C-10-CHO-PteGlu) was added and again equilibrated for 15 min. After cooking for 10 min, 200 μL of rat serum and 900 μL of the chicken pancreas were added, and the samples were shaken overnight in a 37^°^C water bath (GFL, Burgwedel, Germany). The second extraction day started with cooking the samples for 10 min at 100°C, followed by adding 10 mL acetonitrile (HPLC grade) and centrifugation (4,000 rpm, 4°C, 20 min) (Centrifuge 5810R, Eppendorf AG, Hamburg, Germany) of the samples. The supernatants were purified by solid-phase extraction with Strata^®^ strong anion exchange (SAX) cartridges (quaternary amine, 500 mg, 3 mL) and measured by LC-MS/MS at least in duplicate.

### Instrumental conditions

#### High performance liquid chromatography with diode-array detection

The standard solutions of the unlabeled compounds were quantified on a Shimadzu HPLC/DAD system (Shimadzu, Kyoto, Japan) using a reversed-phase column (C18 EC, 250 × 3 mm, 5μm, 100 Å, precolumn: C18, 8 × 3 mm, Machery-Nagel, Düren, Germany) for separation. The mobile phase consisted of 0.1% acetic acid in water (A) and methanol (B) delivered as a binary gradient. The gradient started at 10% B for 7 min and increased slowly to 50% B in 14 min. Next, it increased linearly to 100% B in 2 min, was held for 1 min, and decreased in 2 min to 10% B, where it was held for 9 min. The injection volume was 10 μL, and the column temperature was set to 25°C. For quantification, a wavelength of 272 nm was used for H_4_folate and 10-CHO-PteGlu and 290 nm for 5-CH_3_-H_4_folate and 5-CHO-H_4_folate.

#### Liquid chromatography–mass spectrometry

The quantification of the samples was performed on a Shimadzu Nexera X2 UHPLC system (Shimadzu, Koyoto, Japan) with a Raptor™ ARC-18 (2.7 μm, 100 × 2.1 mm, Restek, Bad Homburg, Germany) column and a Raptor™ EXP guard column (2.7 μm, 5 × 2.1 mm; Restek, Bad Homburg, Germany). The flow rate was 400 μL/min, the column temperature was 30°C, and the injection volume was 10 μL. A binary gradient with a mobile phase consisting of 0.1% formic acid in water (A) and 0.1% formic acid in acetonitrile (B) was used. The gradient started at 3% B for 1 min increased to 10% B in 2 min and was held for 2.5 min. Afterward, it increased to 15% B in 5 min, increased linearly to 50% B in 1 min, was held for 1 min before it decreased again to 3% B in 1 min, and equilibrated for 4 min.

The LC was interfaced with a triple quadrupole ion trap mass spectrometer (LCMS-8050, Shimadzu, Kyoto, Japan). It operated in a positive ESI ionization mode for all analytes and in a multiple reaction monitoring (MRM) mode. The used MS parameters were 300^°^C as interface temperature, 250^°^C as DL temperature, 400^°^C as heat block temperature, 3 L/min as nebulizer gas flow, 10 L/min as heating gas flow, 10 L/min as drying gas flow, 4 kV as interface voltage, and 270 kPa as CID gas.

### Statistical evaluation

All results are normally distributed using the David test at a significance level of α = 0.05. The samples were extracted five times and injected at least two times. The Dean and Dixon test excluded outliers at a level of significance of α = 0.05. The mean and standard deviation were calculated from at least three values. The only exceptions were the bramble leaves and the hay samples, which were only calculated from values of two extractions due to insufficient homogenization. As the data of the samples are only indicative and not representative with respect to the sample number and sample size, we did not perform statistical tests for significant differences. The data of the preliminary study are intended to provide the first insight into the folate content of currently used insects.

## Results

**FIGURE 2:**
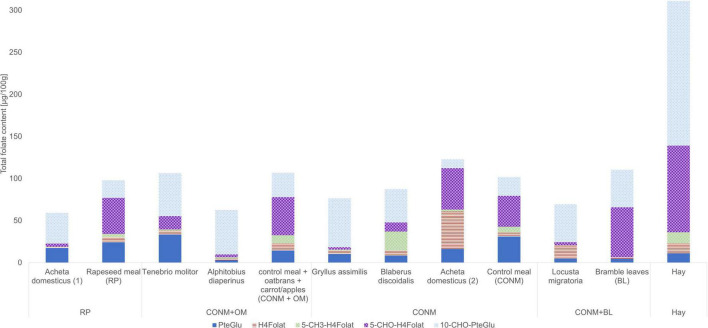
Vitamer distribution of the folate content in the insects and the feed, calculated as PteGlu (μg/100 g of fresh weight).

**FIGURE 3:**
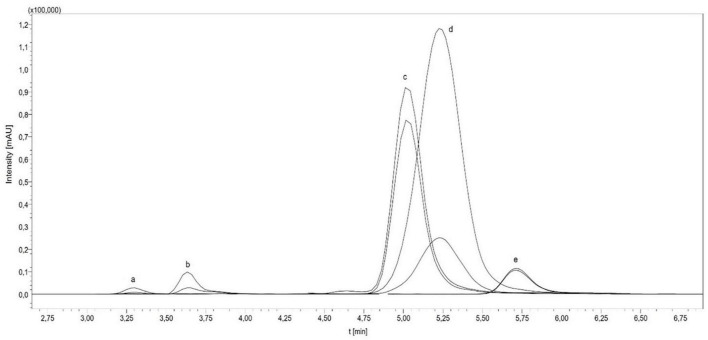
LC-MS/MS chromatogram of *Acheta domesticus* [(a) H_4_folate, [^13^C_5_]-H_4_folate; (b) 5-CH_3_-H_4_folate, [^13^C_5_]-5-CH_3_-H_4_folate; (c) 10-CHO-PteGlu, [^13^C_5_]-10-CHO-PteGlu; (d) 5-CHO-H_4_folate, [^13^C_5_]-5-CHO-H_4_folate; (e) PteGlu, [^13^C_5_]-PteGlu].

In the study, the total folate content and the vitamer distribution of six different insect species and the feedstuff were analyzed. Figure 2 shows the vitamer distribution of all samples, and Tables 1, 2 list the folate contents in fresh weight. Furthermore, Figure 3 shows the vitamer distribution of the *A. domesticus* sample as an example of a typical LC-MS/MS chromatogram.

**TABLE 1:** Total folate content and vitamer distribution in insect samples, calculated as PteGlu [μg/100 g of fresh weight].

Tested sample	PteGlu [μ g/100 g]	H_4_folate [μ g/100 g]	5-CH_3_-H_4_folate [μ g/100 g]	5-CHO-H_4_folate [μ g/100 g]	10-CHO-PteGlu [μ g/100 g]	Total folate (fresh) [μ g/100 g]	Feed
*Acheta domesticus*—adults (1) (frozen)	17.5 ± 4.71	0.76 ± 1.00	0.61 ± 0.82	3.92 ± 0.53	36.3 ± 4.72	59.1 ± 6.50	Rapeseed meal (RP)
*Acheta domesticus*—adults (2) (lyophilized)	16.4 ± 5.15	44.1 ± 24.0	2.51 ± 0.70	49.3 ± 4.41	10.4 ± 3.71	123 ± 29.8	Control meal (= chicken feed) (CH)
*Gryllus assimilis*—adults	11.0 ± 1.70	3.60 ± 2.29	<LOQ^a^	2.59 ± 0.24	63.3 ± 10.5	80.4 ± 10.0	Control meal (= chicken feed) (CH)
*Blaberus discoidalis*—large nymphs	7.58 ± 3.20	5.49 ± 3.18	20.4 ± 6.11	10.8 ± 1.08	37.4 ± 4.83	81.6 ± 13.4	Control meal (= chicken feed) (CH)
*Locusta migratoria*—adults	4.78 ± 1.26	10.4 ± 7.56	<LOQ^a^	4.60 ± 1.80	43.4 ± 2.53	63.2 ± 7.96	Control meal (= chicken feed) (CH) + Bramble leaves (BL) + Hay
*Tenebrio molitor*—worms	28.1 ± 6.53	4.39 ± 1.94	1.62 ± 0.83	15.8 ± 3.38	51.2 ± 7.37	101 ± 9.97	Control meal + oat bran + carrot/ apples (CH + WB)
*Alphitobius diaperinus*—worms	3.00 ± 2.27	1.87 ± 0.24	1.57 ± 0.11	3.28 ± 1.20	52.8 ± 2.86	62.5 ± 2.11	Control meal + wheat bran + carrot/ apples (CH + WB)

^a^<LOQ: Below the limit of quantification (5-CH_3_-H_4_folate: 0.51 μg/100 g).

**TABLE 2:** Total folate content and vitamer distribution in feedstuffs, calculated as PteGlu [μg/100 g].

Tested sample	PteGlu [μ g/100 g]	H_4_folate [μ g/100 g]	5-CH_3_-H_4_folate [μ g/100 g]	5-CHO-H_4_folate [μ g/100 g]	10-CHO-PteGlu [μ g/100 g]	Total folate (fresh) [μ g/100 g]
Rapeseed meal (RP)	24.3 ± 15.9	6.10 ± 3.76	3.71 ± 2.66	42.9 ± 13.4	21.0 ± 1.21	98.0 ± 3.04
Control meal (= chicken feed) (CH)	31.0 ± 9.14	5.04 ± 2.53	6.59 ± 2.25	36.7 ± 5.67	22.4 ± 2.55	102 ± 2.03
Control meal + oat bran + carrot/apples (CH + WB)	14.4 ± 12.8	8.75 ± 2.70	9.45 ± 6.42	45.3 ± 10.9	29.0 ± 3.25	107 ± 3.69
Bramble leaves (BL)	4.63	1.90	<LOQ^a^	59.3	44.7	110
Hay	11.1	11.9	13.2	103	172	311

^a^<LOQ: Below the limit of quantification (5-CH_3_-H_4_folate: 0.51 μg/100 g).

*Acheta domesticus* samples fed with a rapeseed meal had a total folate content of 59.1 μg/100 g and differed from the content of *A. domesticus* fed with the control meal (123 μg/100 g). *A. domesticus* thus represents the highest and the lowest content investigated at the same time due to different feeding conditions and the way of subsequent processing (freezing vs. lyophilization). *L. migratoria* and *A. diaperinus* showed a similar folate content of 63.2 and 62.5 μg/100 g, respectively. Also, the. *B. discoidalis* and *G. assimilis* showed results in a similar range of 81.6 μg/100 g and 80.4 μg/100 g, respectively. In *T. molitor*, the second highest value of 101 μg/100 g was detected. Furthermore, the feed showed a total folate content between 98.0 μg/100 g and 110 μg/100 g, apart from hay, which had an outstanding high folate content of 311 μg/100 g. This can be explained by folate concentration due to the low water content in hay compared to the other feed.

Regarding the folate vitamer distribution, the samples were quite diverse: all samples contained 3 to over 31 μg/100 g of the synthetic form PteGlu, and the content of 10-CHO-PteGlu was generally high except for one *A. domesticus* adult (2) sample. The samples were surprisingly low in 5-CH_3_-H_4_folate, except *B. discoidalis* large nymphs, which contrasts with plant samples that are generally high in this vitamer. 5-CHO-H_4_folate showed huge differences among the species and also within the insect species of *A. domesticus*. The latter species also revealed a large variation in their total folate content and vitamer distribution, with one sample particularly high in H_4_folate and other samples showing high contents of 5-CHO-H_4_folate.

## Discussion

Although insects are an extremely variable group of Arthropods, whose diversity is estimated to be more than six million species, only about 2,100 of them are known to be consumed by humans worldwide (36). A majority of edible insects are currently still harvested from the wild, which will become unsustainable when entomophagy would become promoted (1). Also, the food safety concerns are higher in wild insects. The artificial rearing of insects is thus the unique option ensuring a sufficient amount of high-quality insect biomass yield with minimal environmental impacts. However, only a few species are suitable for this purpose, when a rapid life cycle, high biomass gain, no special life requirements (e.g., diapause), and easy manipulation are the main issues (37). From this point of view, all the analyzed insects are belonging to such favorable species. Crickets, locusts, and mealworms are currently produced for human consumption in many parts of the world, including Europe (1). On the one hand, cockroaches are often raised by institutions as experimental organisms, for medical purposes, or as feed for insectivorous hobby animals. Their reputation as noxious household pests is very bad, and hardly anybody could expect people would adopt them into their diet as whole insects. On the other hand, our recent survey revealed no special preference in willingness to eat insects in their hidden form (38, 39). Therefore, we also decided to add one cockroach species to the list of tested species. Food intake and nutrient preferences are also known to be influenced by life stages. In this study, we harvested the insect that had the highest individual weights. While crickets and locusts reach the highest weights in the adult stage, the cockroaches gain the highest weight in the subadult stage (38). Similarly, mealworms were harvested when first immobile instars were observed in order to prevent weight loss during pupation (40).

Vitamins are known to be highly tied with diet composition (13, 20). To prevent these interferences, the efforts to keep insects, in the same manner, using the same feed would be optimal. However, the life requirements of the tested species varied, so this scenario could not be fully followed. The crickets and cockroaches were supplied by the chicken feed and water gel only. Being herbivorous, *L. migratoria* requires high fiber content in the diet. To meet this requirement, bramble leaves and hay were supplemented with the chicken feed. Since the substrate mealworms live in also serve as their feed, it is necessary to add wheat bran to the chicken feed for a increased airiness and enable crawling. Also, the water gel is not suitable for mealworms due to the increased risk of mold occurrence in the substrate. In the case of crickets, cockroaches, and locusts, water soaked in the gel was supplied on Petri dishes to prevent degradation of feed.

The results of the SIDA in the insects revealed that total folate contents range between 60 and 123 μg/100 g (Table 1), which is significantly higher than that in other samples of animal origin like pork or beef, with folate content below 10 μg/100 g (41). However, when comparing the insect folate content with samples of plant origin, the content is in a similar range for green vegetables (42) or fruits like strawberries, feijoa, passion fruit, papaya, or mango (43, 44).

Considering previous studies on folates in insects, four different insect species (mealworms, crickets, superworms, and waxworms) were analyzed for their folate content by microbiological assay (19). The results range between 61 and 155 μg/100 g, which agrees with the results in the present study. Due to different feeding and growth stages, the comparison is only possible to a limited extent, but the content of mealworms, which are at least in the same growth stage, is 107 μg/100 g (19), which is very similar to the content given here. An earlier publication also showed similar results between 44 and 157 μg/100 g for different insect species (45). In a review, the limited data available on the vitamin content of edible insects was pointed out (46). However, the value given for mealworm larvae of 137 μg/100 g is also in agreement with our results. More recently, we reported on the folate content in green ants (*Oecophylla smaragdina*) consumed by indigenous people in Australia (47). Total folate amounted to 200–310 μg/100 g dry weight, which corresponds to 72 μg/100 g fresh weight in mean when considering the water content in the samples. The green ant samples were considerably high in 10-CHO-PteGlu, which is in line with almost all insect samples investigated in this study. In order to shed light on the reasons for the high variations, we also investigated the feed used for growing the insects. The folate analyses of the feed (Table 2) showed total folate contents of generally around 100 μg/100 g, with the exception of hay showing the highest content of 311 μg/100 g. Interestingly, all feedstuffs were high in 5-CHO-H_4_folate and 10-CHO-PteGlu. In particular, the rapeseed meal and the control meal contained significant amounts of PteGlu, which indicated that these feeds were supplemented with this synthetic vitamer. The rather low contents of 5-CH_3_-H_4_folate and H_4_folate may be due to the lability of these vitamers and to degradation during processing and storage.

When comparing folate contents and vitamer distributions of the feedstuff and the respective insects being fed, only few interrelations appear to be consistent (Figure 2). Almost all insect samples were low in 5-CH_3_-H_4_folate and H_4_folate, which is in line with the respective low concentration in the feedstuffs. The only exceptions are *A. domesticus* adults (2) and *B. discoidalis* large nymph sample for H_4_folate and 5-CH_3_-H_4_folate, respectively. Some correlation can be found between the significant content of PteGlu in many insect samples and the PteGlu content in most of the underlying feedstuff. Here, it appears that this synthetic oxidized vitamer is not efficiently metabolized into the reduced vitamer forms, which are supposed to be the physiologically active ones. Obviously, the feed does not appear to be the sole determinant of the vitamer present in the organism. Other parameters, such as the species or the physiological state, appear to have a higher impact.

Regarding their total folate content, insects appear to be promising sources for folates in general. However, one has to consider how many insects may be consumed during a meal and to what amount insect meal can contribute to a composite food. Taking into account the restriction of insect powder as an ingredient to replace cereal flour at about 10%, a maximum daily consumption of 20 g of dry insect powder can be assumed. From these estimations, a total contribution of insects (e.g., *A. domesticus*—adults) to the recommended daily intake of 400 μg/100 g according to the WHO and the United States recommendation, 7% can be calculated. Considering the reference values of the German, Austrian, and Switzer Nutrition Society (D-A-CH reference values) of 300 μg/100 g, 10% of the daily intake can be achieved.

From our studies, an impact of the feed on insect folate can be assumed. A carryover of the synthetic PteGlu into the insects without major metabolism appears obvious, but the correlations for the other vitamers and the total folate, in general, are still unclear. To shed light on these correlations along with the impact of the species, the age and physiological state of insects require further research. In addition, the effect of fasting, the way of killing, and further processing such as drying and culinary treatment (baking, frying, roasting, etc.) could also be important and could change the total folate content and vitamer distribution in each individual insect species available on the market.

## Data availability statement

The original contributions presented in this study are included in the article/supplementary material, further inquiries can be directed to the corresponding author/s.

## Author contributions

MK, LK, and DP reared and prepared the insect samples. NW and FS performed the experiments and analyzed the data. MK, LK, MR, and NW wrote the manuscript. All authors contributed to the article and approved the submitted version.
